# Hepatitis B sero-prevalence among hematology patients: importance of Anti-HbcAb and efficiency of antiviral prophylaxis

**DOI:** 10.4314/ahs.v22i3.60

**Published:** 2022-09

**Authors:** Funda Ugur Kantar, Selda Kahraman, Gulfem Ece, Seckin Cagırgan

**Affiliations:** 1 Cigli State Hospital, Department of Gastroenterology, Izmir, Turkey; 2 Medicalpark Izmir Hospital, Department of Hematology, Izmir, Turkey; 3 Izmir University of Economics, School of Medicine, Medicalpark Izmir Hospital, Department of Medical Microbiology, Izmir, Turkey; 4 Medical park Izmir Hospital, Department of Hematology, Izmir, Turkey

**Keywords:** Hepatitis B, Hematologic Malignancy, stem cell transplantation

## Abstract

**Objectives:**

Hepatitis B infection is an important problem in immune suppressed patients. Anti HbcAb is an important marker that shows past exposure to virus. In this study, we retrospectively searched HBV serology among the patients who had Bone Marrow Transplantation (BMT) or chemotherapies (CT) at Medicalpark Izmir Hospital Bone Marrow Transplantation Unit; changes in viral parameters throughout therapy; and tried to find the efficiency of antiviral prophylaxis.

**Methods:**

We retrospectively evaluated the viral parameters; HbsAg, Anti HbsAb, Anti Hbc IgG, HbeAg, Anti Hbe Ab, HBV DNA, HCV RNA which were carried out before BMT and CT. We grouped the patients as latent HBV infection and inactive carriers. Started antiviral treatment as prophylaxis, monitored the changes in serological parameters and defined HBV related situations.

**Results:**

A total of 584 patients were evaluated retrospectively. Twenty patients were having latent HBV infection. Ten patients were inactive carriers of HBV. In post-transplant period, the patients were screened for 11 months (1–38 months). None of the patients experienced HBV activation during follow period.

**Conclusion:**

The best approach in HbcAb positive patients with planned immunosuppressive treatment is the use of anti-viral agents before immune suppression and close monitoring of the patients HBV-related markers.

## Introduction

Hbv infection is one of the most widely seen viral infections. About 350 million of people worldwide have the diagnosis of chronic hepatitis B^1^. Each year; an estimated one million people die due to complications of chronic HBV infection, like cirrhosis, end-stage liver disease and hepatocellular carcinoma^2^. Turkey is an intermediate-endemic country for HBV infection, with the prevalence of HbsAg and anti-HBs 4.0% and 31.9%, respectively^3^. The prevalence alters widely with geographic regions, like 2.3% in Aegean and 7.3% in Southeastern Anatolia^1^. In the same study, isolated anti-HBc positivity was 4.6% whereas anti-HBs positivity with anti-HBc positivity was 22.0%. These patients with anti-HBc positivity represent the ones with past-exposure to HBV, and the importance of them is the high-risk of reactivation in cases of immune-suppression, like cancer chemotherapy, immune-suppressive or biological treatment, solid organ transplantation or bone marrow transplantation, since there is yet no totally-curative treatment for HBV infection^1^.

Bone marrow transplantation (BMT) has become an important and curative therapy for hematological disorders but also creates a high risk of morbidity and mortality by causing viral reactivation in patients who had met the virus before immune suppressive treatments. The same risk continues with the use of donors who are Anti-Hbc positive. Studies about the mechanism of HBV reactivation after immune suppression points a rebound increase in number of lymphocytes after stopping immune suppression which results in destruction of infected hepatocytes causing hepatitis. Cytokine analysis showed a decrease in CD4-CD25 T-regulatory cell numbers and an increase in antigen specific cytotoxic T-lymphocytes responsible for liver injury^4^.

Reactivation of HBV can initiate a cascade of events from hepatitis to acute liver failure and death. HBV reactivation may also result in discontinuation of hematological treatment. Proper treatment of HBV infection should be given as early as possible, but there may be problems about the recognition of reactivation since these patients are prone to drug induceliver diseases and other forms of viral hepatitis which can cause delay in diagnosis. The ability of HBV to persist in latent replicative form despite the signs of viral clearance may also cause confusion^5^. In cases of immune suppression, for patients carrying a high risk of HBV reactivation, prophylactic oral antiviral treatment is highly recommended to prevent these situations.^6^,^7^

In this study, our aim was to evaluate the seroprevalence of HBV among hematology patients, the changes in viral parameters after chemotherapies and bone marrow transplantation and find the efficiency of antiviral prophylaxis given according to the serological parameters.

## Materials and Methods

### Subjects

In this study, we retrospectively searched HBV serology among the patients who had BMT or CT between the years 2012 and 2016, at Izmir University of Economics, Medical park Izmir Hospital Bone Marrow Transplantation Unit, changes in viral parameters throughout therapy; and try to find the efficiency of antiviral prophylaxis given according to the serological parameters. We evaluated the viral parameters; HbsAg, Anti HbsAb, Anti Hbc Ab, HbeAg, Anti Hbe Ab and HBV DNA; which are the assays routinely carried out before BMT and CT. These assays take part in pretreatment protocol and are not specific for this study. Among patients with a positive serology for HBV; the ones with the diagnosis of chronic HBV infection who were on antiviral treatment were not included. We grouped the patients as latent HBV infection (AntiHbcAb+, Anti Hbs-, HbsAg-, HBV DNA -) and inactive carriers (HbsAg+ , HBV DNA+, ALT:normal ) and monitored the efficiency of antiviral prophylaxis in these groups. In this study, we also documented changes in liver function tests and searched the signs of HBV activation.

Among 584 patients, we observed changes of viral parameters only in 3 patients mentioned above. No viral parameter change has been detected at the remaining.

## Discussion

Hepatitis B is a global health problem, affecting 6% of the whole world population with a large regional variation of prevalence^8^. HBV reactivation is an emerging complication of the virus by the growing use of immune suppressive therapies and organ transplantation. Today BMT has become standard therapy for most of hematological malignancies. However, Immune suppression protocols may activate HBV not only in HbsAg postive patients but also in patients with past exposure to virus that can be identified with Hepatitis B core antibody testing (anti-Hbc Ab). In our retrospective study, we evaluated 584 patients treated with BMT or CT and found 20 patients with latent infection and 10 patients as inactive HBV carriers before their hematological treatments. Our study showed the protective effect of antiviral prophylaxis given before immune suppressive treatments.

Cakar et al reported 5 patients with HBV reactivation and 2 patients with acute hepatitis B among 197 patients who underwent hematopoietic stem cell transplantation. They did not give prophylaxis in patients with anti -Hbc positive and Hbs ag negative patients and observed no HBV reactivation in this group^9^. Vigano did not use pretreatment prophylaxis and reported HbsAg seroreversion of 12% in patients with HbsAg negative/anti HbcAb positive^10^. Without prophylaxis, the ratio may be as high as 20% among patients with autologous SCT and 9.1% among allogeneic SCT^11^.

Mikulska et al reported HBV reactivation ratio of 10% in patients who were HbsAg negative but HbcAb positive before allogenic hematopoietic stem cell transplantation^12^. They did not use prophylaxis for HBV. There are also some other studies reporting HBV reactivation in the range of 11–29% whose serological markers were negative for HbsAg but positive for AntiHbcAb before BMT^13^–^17^.

In our study, we continued antiviral prophylaxis up to one year after cessation of immune suppression, and it was effective since we did not observe any patients with HBV reactivation. There are different proposals like the use of antiviral prophylaxis more than 24 months^18^. Long-term prophylaxis may be used selectively by anticipating the high-risk patients. Another approach is close monitoring of HBV DNA and use of antivirals in cases of reactivation^18^. Despite the use of antiviral agents, HBV reactivation may be fatal, and the risk may increase up to 12%^11^–^19^–^20^. Studies point out the importance of having chronic onco-hematological disease, long duration of immune suppression and variety of chemotherapeutics, low antiHbs titre and even loss of anti-hbs and type of BMT either autologous or allogeneic, as factors increasing the risk of HBV-reactivation^10^,^11^,^18^.

In 3 of our patients, that were HbsAg and anti-hbc negative before BMT, we detected increase in serum ALT levels by routine controls after transplantation. These patients were having acute HBV infection, with Hbsag and anti-HbcIgM positivity. Rapid and effective antiviral treatment enabled us to control the infection. Close monitoring of the patients through elevated ALT levels, which was the warning sign, made us be aware of the problem.

Since the serological tests of these 3 patients were negative for HBV before BMT, the only explanation of the situation could be seronegative occult HBV infection either in the donor or in the recipient. Occult HBV (OBI) infection can be defined as the persistence of HBVgenomes in the liver (with detectable or undetectale HBV DNA in the serum) in individuals testing as negative HBsAg and positive / negative anti-HbcIgG.^21^. OBI is an important condition in hematological diseases because development of an immune suppressive status mainly by immunotherapy or chemotherapy can induce OBI reactivation and cause development of acute and often severe hepatitis^22^. HBV can also be transmitted through blood transfusion and liver transplantation causing classic forms of hepatitis B in newly infected individuals. Although OBI is significantly associated with the presence of anti-HBV antibodies (anti-HBc and anti-HBs antibodies), more than 20% of occult-infected individuals are negative for all HBV serum markers^23^. In seronegative OBI, only HBV DNA is detectable in serum or liver tissue, but anti-HBcIgG/anti-HBsIgGs are negative in serum^24^. Both blood transfusion and organ transplantation increases the risk of OBI transmission. There are several studies about OBI in the literature. In Hong Kong, the prevelance of occult HBV was found to be 15.3% among HbsAg negative stem cell donors^25^. In Taiwan, the prevalence was 0.11% in blood donors^26^. In Egypt, it varied from 4.1% to 26.8% in hemodialysis patients^27^,^28^. In Iran, the prevalence of OBI has been reported as 2 in 50000 in blood donors and 14% in cryptogenic liver cirrhosis patients, while the prevalence of seropositive OBI was 2.27%^29^–^31^. Thus, OBI is an emerging problem especially in endemic areas and HBV transmission from an OBI donor or recipient with OBI is a well known cause of HBV infection after immune supression periods, which forms the basis for antiviral prophylaxis^32^.

Vaccination of both HBV naive donor and recipient before BMT may decrease the risk of acute HBV infection especially in intermediate and highly endemic regions^33^. İmmunity to HBV gained by vaccination can disappear after transplantation and this may reach to %57, but interestingly there are reports of seroconversion in patient who are HbsAg positive before but became antihbs antibody posivite after transplantation from a HBV-immune donor^33^. In these cases, it is thought to be due to adoptive passage of this HbsAg specific cytotoxic T lymphocytes from donor to recipient. The immunity may either be from vaccination or natural infection^19^.

As a conclusion, patients undergoing BMT or CT should be checked for viral serological markers before hematological treatments. It is important to be aware of the complications of HBV in these immune suppressed patients. In our opinion, the best approach in inactive HBV carriers and HbsAg negative, HbcAb positive patients with planned immune suppressive treatment is the use of anti-viral agents before immune suppression. Antiviral treatment is safe and effective in preventing HBV related complications. Our study also showed that serological markers such as HbsAg, Anti Hbs and HbcAb may not be adequate for detection of occult HBV infection. Close monitoring of the patients both by clinical and laboratory parameters is the key point for being aware of complications of HBV.

## Figures and Tables

**Table 1:** Patient Characteristics

	Inactive carriers	Latent infection
	n:10	n:20
**Median age(years)**	51,5 (27–70)	64,5 (33–82)
**Gender F/M**	2/8 (20% / 80%)	5/15 (25% / 75%)
**Diagnosis**		
Multiple myeloma	3 (30%)	8 (40%)
Non-hodgkin lymphoma	4 (40%)	6 (30%)
Acute myeloid leukemia	2 (20%)	4(20%)
Acute lymphocytic leukemia		1 (5%)
Hodgin lymphoma		1 (5%)
Chronic myeloid leukemia	1 (10%)	
**Type of transplantation**		
Autologous	6 (60%)	12(60%)
Allogenic	1 (10%)	2 (10%)
**Chemotheraphy**	3/10 (30%)	6/20 (30%)
**GVHD prophylaxis**	0	0
**Anti-viral prophylaxis**		
Lamivudin	8 (80%)	16 (80%)
Tenofovir	2 (20%)	1 (5%)
None	0	2 (10%)
**HBV reactivation**	0	0
**Death**	3/10 (30%)	5/20(25%)
Primary disease	3 (30%)	5 (25%)
Others	0	0
